# Drug delivery with a pH-sensitive star-like dextran-graft polyacrylamide copolymer

**DOI:** 10.1039/d2na00353h

**Published:** 2022-10-10

**Authors:** Anna Grebinyk, Svitlana Prylutska, Sergii Grebinyk, Stanislav Ponomarenko, Pavlo Virych, Vasyl Chumachenko, Nataliya Kutsevol, Yuriy Prylutskyy, Uwe Ritter, Marcus Frohme

**Affiliations:** Division Molecular Biotechnology and Functional Genomics, Technical University of Applied Sciences Wildau Hochschulring 1 15745 Wildau Germany mfrohme@th-wildau.de; National University of Life and Environmental Science of Ukraine Heroiv Oborony Str., 15 03041 Kyiv Ukraine; Taras Shevchenko National University of Kyiv Volodymyrska Str., 64 01601 Kyiv Ukraine; Institute Charles Sadron 23 Rue du Loess 67200 Strasbourg France; Technical University of Ilmenau, Institute of Chemistry and Biotechnology Weimarer Str., 25 98693 Ilmenau Germany

## Abstract

The development of precision cancer medicine relies on novel formulation strategies for targeted drug delivery to increase the therapeutic outcome. Biocompatible polymer nanoparticles, namely dextran-*graft*-polyacrylamide (D-*g*-PAA) copolymers, represent one of the innovative non-invasive approaches for drug delivery applications in cancer therapy. In this study, the star-like D-*g*-PAA copolymer in anionic form (D-*g*-PAAan) was developed for pH-triggered targeted drug delivery of the common chemotherapeutic drugs – doxorubicin (Dox) and cisplatin (Cis). The initial D-*g*-PAA copolymer was synthesized by the radical graft polymerization method, and then alkaline-hydrolyzed to get this polymer in anionic form for further use for drug encapsulation. The acidification of the buffer promoted the release of loaded drugs. D-*g*-PAAan nanoparticles increased the toxic potential of the drugs against human and mouse lung carcinoma cells (A549 and LLC), but not against normal human lung cells (HEL299). The drug-loaded D-*g*-PAAan-nanoparticles promoted further oxidative stress and apoptosis induction in LLC cells. D-*g*-PAAan-nanoparticles improved Dox accumulation and drugs’ toxicity in a 3D LLC multi-cellular spheroid model. The data obtained indicate that the strategy of chemotherapeutic drug encapsulation within the branched D-*g*-PAAan nanoparticle allows not only to realize pH-triggered drug release but also to potentiate its cytotoxic, prooxidant and proapoptotic effects against lung carcinoma cells.

## Abstract 2

The development of precision cancer medicine relies on novel formulation strategies for targeted drug delivery to increase the therapeutic outcome.

## Graphical abstract

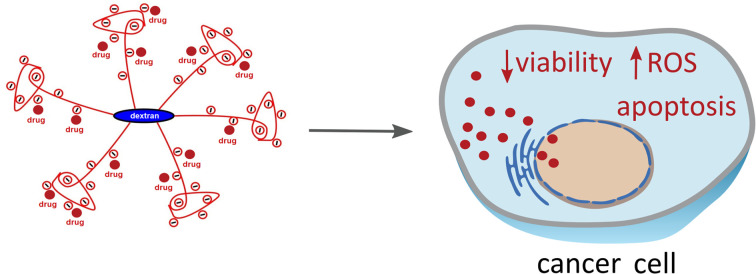

## Introduction

Lung cancer is a leading cause of cancer-related death, responsible for 2.2 million new cases and 1.8 million deaths in 2020 worldwide.^1^ The risk of lung cancer, strongly associated with smoking and air pollution, is anticipated to continue growing. Nowadays, traditional antitumor chemotherapy is used for treatment of most cancers, based on the application of small toxic chemotherapeutic molecules that interact with DNA molecules, modify them and induce cell death in cancer tissues.^2,3^ However, conventional chemotherapeutics suffer from a number of limitations, including poor solubility, stability and short blood circulation that result in low drug bioavailability and selectivity. Moreover chemotherapeutic drugs possess high toxicity and damage not only cancer, but also healthy cells, producing diverse side effects.^4,5^ The use of modern nanobiotechnology could potentially solve this problem and be more efficient in comparison with traditional anticancer chemotherapeutic drugs. Nanobiotechnology can provide targeted delivery of the drug to cancer tissue and reduce its systemic toxicity. Also it gives a possibility to control the drug concentration, prolong blood circulation and release it targetly on the site of interest.^5–9^

Polymeric nanocarriers offer remarkable progress in the anticancer treatment of disease in the near future.^10–13^ A polymer nanocarrier loaded with anticancer drugs overcomes many disadvantages associated with conventional drug delivery. A large surface to volume ratio, encapsulation of hydrophobic molecules, optimization and control of drug dosage, slow and sustained release, targeting of the specific pathological tissues provide major advantages for such systems. Thus, polymers can be classified as smart drug delivery systems which can provide superior biocompatibility and biodegradability and easy preparation.^14–16^ Moreover, the polymer nanocarrier can be pH- or thermosensitive, that expands the use of such nanosystems for solving the specific task of targeted drug delivery in cancer treatment.^16–18^

Recently a star-shaped dextran-graft polyacrylamide (D-*g*-PAA) copolymer has been exploited as a potential nanocarrier for photodynamic therapy^19^ and chemotherapy.^20^ It was proved that the branched star-like copolymer was efficient for the encapsulation of anticancer drugs. The copolymer was absorbed by macrophages (murine macrophage cell line) and was not cytotoxic.^21^ A polymer-cisplatin (Cis) nanosystem yielded a dose-dependent decrease in the viability of K-562 (human chronic myelogenous leukemia) and U-937 (human histiocytic lymphoma) cell lines at different concentrations from 0.1 to 10 μg mL^−1^. Thus, the polymer-Cis nanosystem decreased cell viability to about 22% and 39% at 5 μg mL^−1^, respectively.^21^

Branched copolymers have been proven to be capable of stabilizing potentially unstable nanosystems.^22^ The number of variable parameters affecting the macromolecule structure of branched polyelectrolytes increases substantially, as compared with their nonionic analogs.^23^ Conformational changes leading to changes in the size and compactness of macromolecules are additionally controlled by the pH and the ionic strength of the environment, thus expanding the range of their usage as smart materials for pH-responsive targeted drug delivery. A pH-triggered delivery strategy targets the acidic intracellular organelles and extracellular microenvironment of solid tumours.^14,24,25^ Thus, pH-responsive polymer nanoparticles can offer a powerful strategy to design smart therapeutic delivery systems.

Here we discuss an application of the anionic analogue of the D-*g*-PAA copolymer, namely D-*g*-PAAan as a targeted nanocarrier for anticancer drugs doxorubicin (Dox) and Cis, as well as compare their cytotoxic activity *in vitro* towards human and mouse lung carcinoma cells (A549 and LLC) and normal human lung cells (HEL299). For this, first, the size distribution and pH-dependent drug release were assessed for the synthesized D-*g*-PAAan-Dox and D-*g*-PAAan-Cis followed by cell-based evaluation of its effects on cell viability and morphology, reactive oxygen species (ROS) generation levels and caspase 3/7 activation in the lung cells of different origins.

## Results and discussion

### Dynamic light scattering (DLS) study

**Fig. 1:**
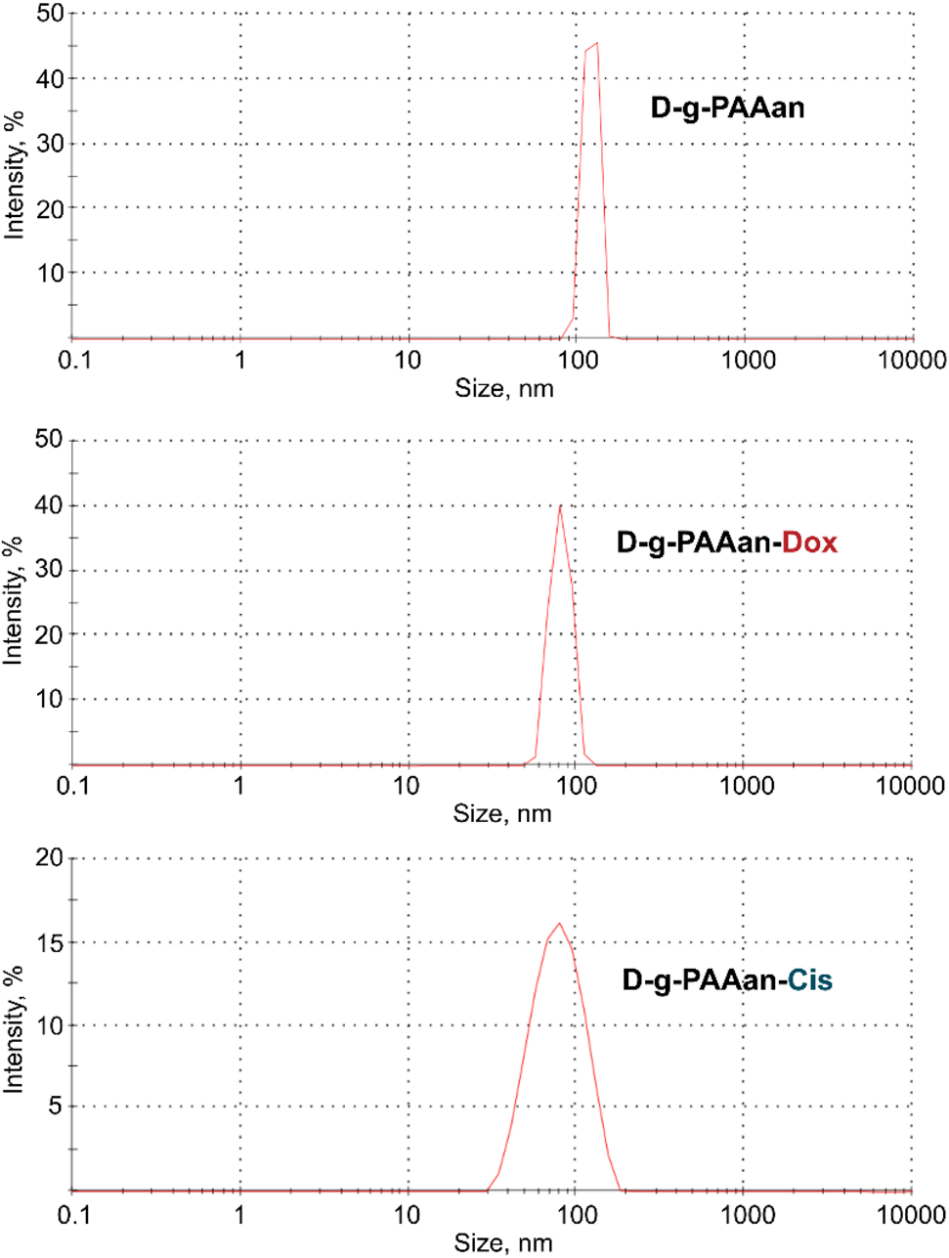
Hydrodynamic size (diameter, nm) of free, Dox- and Cis-loaded D-*g*-PAAan particles; intensity in %: percentage of all scattered light intensity.

The DLS data on the hydrodynamic diameter distribution of the D-*g*-PAAan nanoparticle aqueous systems quantified their size to be 120 nm with a polydispersity index (PDI) of 0.185 (Fig. 1). Once the nanoparticles encapsulated the drugs a shrinkage was observed in dH_2_O. The size was found to be 80 nm for both Dox- and Cis-loaded polymeric nanoparticles (Fig. 1). This was not surprising, because the functional groups of the polymer were partially bonded with molecules of Dox or Cis caused the negative charge shielding in the grafted chains resulting in shrinkage of the D-*g*-PAAan coil.

The PDI value for the Dox- and Cis-loaded D-*g*-PAAan nanoparticles was estimated at a level of 0.206 and 0.244, respectively, that evidenced the monodisperse size distribution.

The zeta potential is related to the stability of colloidal dispersions. The zeta potential value for the free D-*g*-PAAan, as well as D-*g*-PAAan-Dox and D-*g*-PAAan-Cis aqueous systems was −21.4 mV, −28.6 mV and −23.8 mV, respectively. A high negative surface charge of the individual nanoparticles (or, more strictly, the electrostatic repulsion between the negatively charged aggregates) indicates a very low tendency for them to aggregate over time in an aqueous solution (*i.e.*, a high solute stabilization).

The size distribution and zeta potential of D-*g*-PAAan, D-*g*-PAAan-Dox and D-*g*-PAAan-Cis aqueous systems were stable for 6 months.

To estimate the stability in a cell culture experimental set-up, free D-*g*-PAAan, D-*g*-PAAan-Dox and D-*g*-PAAan-Cis were incubated at 37 °C for 48 h in DMEM supplemented with 1% FBS that mimicked the cell-based assays. At 0, 24 and 48 h the hydrodynamic diameter distribution was measured with DLS (Table 1). The nanoparticle’s hydrodynamic diameter distribution in FBS-supplemented cell culture showed that its size was increased as compared with measurements in dH_2_O. Thus, the free D-*g*-PAAan nanoparticle size distribution was 130 ± 3 nm. The 10 nm-size increase of the free D-*g*-PAAan nanoparticle size distribution suggested a protein corona formation on the surface of the nanoparticle in the FBS-supplemented cell culture DMEM medium. The size distributions of D-*g*-PAAan-Dox and D-*g*-PAAan-Cis nanoparticles were estimated to be around 150 nm. It is known that the structural organization of nanoparticles, the surface of which is charged, is determined not only by the hydrophobic and van der Waals interactions, but also by the electrostatic interactions and therefore significantly depends on the presence of electrolytes in the dissolution medium.^26^ Both, D-*g*-PAAan in aqueous solution and bovine serum albumin (BSA), the major FBS component, have small negative zeta-potential values. However, multi-charged cations in the DMEM medium (Ca^2+^, Fe^3+^ and Mg^2+^) are able to internally cross-link PAAan chains by bonding with COO-groups and significantly decrease the zeta potential of D-*g*-PAAan. As a result, neutrally charged D-*g*-PAAan can interact with BSA in a semi-diluted concentration regime and form aggregates.^27^

**Table 1:** Hydrodynamic size (diameter, nm) of free and Dox- and Cis-loaded D-*g*-PAAan in a 1% FBS-supplemented DMEM medium

Time	0 h	24 h	48 h
D-*g*-PAAan	130 ± 3	132 ± 4	131 ± 3
D-*g*-PAAan-Dox	151 ± 4	154 ± 5	156 ± 4
D-*g*-PAAan-Cis	149 ± 4	147 ± 4	148 ± 5

All nanoparticles had no significant changes in size distributions when measured immediately and during 48 h of incubation under *in vitro* cell culture experimental conditions (Table 1).

The maximum detected stability during prolonged incubation indicated that there was no additional aggregation of the nanoparticles during the prolonged incubation in the FBS-supplemented cell culture medium which confirmed their suitability for *in vitro* studies.

### Drug release

**Fig. 2:**
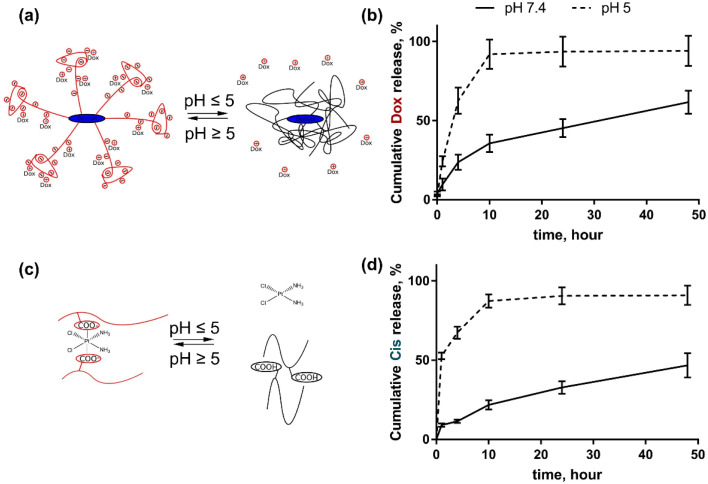
Drug release from D-*g*-PAAan nanoparticles: schematic representation of Dox (a) and Cis (c) release; cumulative release of Dox (b) and Cis (d) during 48 h of incubation at pH 7.4 and 5.0.

The drug release studies from the nanoparticle were conducted in triplicate using dialysis at different pH for 48 h. The concentration of the released drugs was assessed with a HPLC-ESI-MS/MS. The selected pH values were 7.4 and 5.0 that mimicked blood pH and cancer cell pH, which is known to be more acidic. Both drug molecules were released from the nanoparticles at an acidic pH more rapidly than at physiological pH. It can be explained by the fact that at low pH the polyelectrolyte nanocarrier changed its hydrophilic–hydrophobic balance and became more hydrophobic. The COO-groups of the grafted hydrolyzed polyacrylamide chains transform to –COOH ones at pH 5.0. Therefore this leads to the release of encapsulated hydrophilic drugs. The Cis molecule is more hydrophilic in comparison with Dox (Fig. 2).

For this reason at pH 5.0 the cumulative Dox release from D-*g*-PAAan nanoparticles reached 91.9 ± 9.2% at 10 h, whereas at pH 7.4 it was slower reaching the levels of 35.6 ± 5.5 and 61.6 ± 7.2% at 10 and 48 h, respectively (Fig. 2b). Cis was released from D-*g*-PAAan nanoparticles even faster at pH 5.0, thus reaching the level of 52.6 ± 2.2% at 1 h as compared to the level of 9.0 ± 1.0% of released Dox at pH 7.4. At 48 h Cis release was estimated to be 90.9 ± 7.2 and 46.7 ± 7.3% at pH 5.0 and 7.4, respectively (Fig. 2d).

### Cell viability

**Fig. 3:**
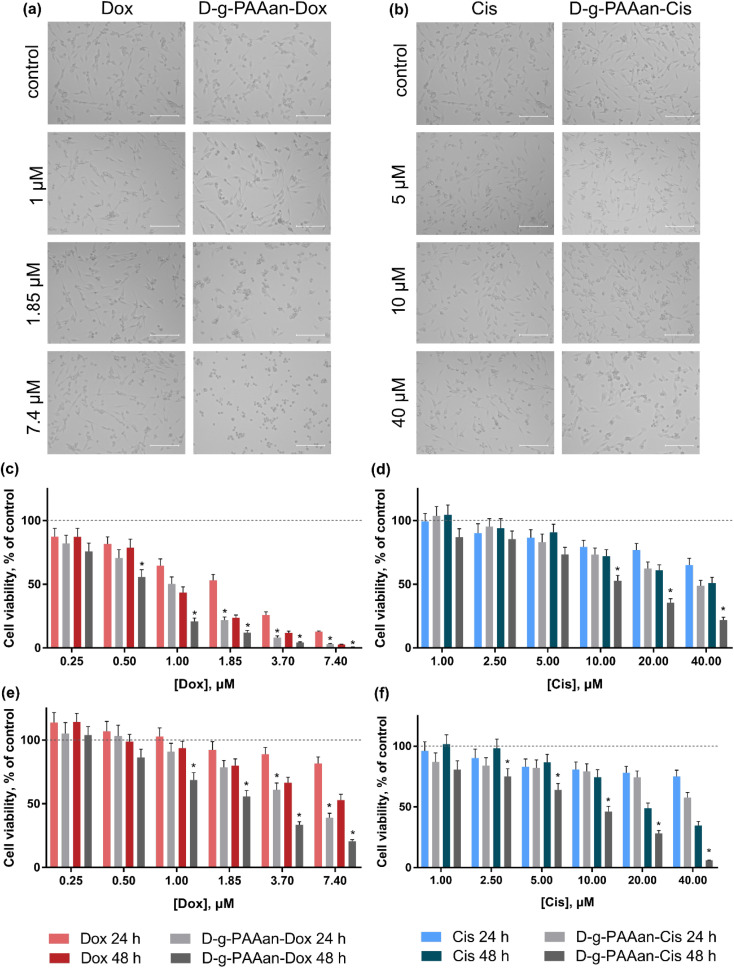
LLC cell viability under the action of free drugs and drug-nanocomplexes: phase microscopy of LLC cells, treated for 24 h with free Dox or D-*g*-PAAan-Dox in Dox-equivalent concentrations (a) or with free Cis or D-*g*-PAAan-Cis in Cis-equivalent concentrations (b), scale bar 150 μm; LLC cell viability after treatment for 24 and 48 h with free Dox or D-*g*-PAAan-Dox in Dox-equivalent concentrations (c) or with free Cis or D-*g*-PAAan-Cis in Cis-equivalent concentrations (d); A549 cell viability after treatment for 24 and 48 h with free Dox or D-*g*-PAAan-Dox in Dox-equivalent concentrations (e) or with free Cis or D-*g*-PAAan-Cis in Cis-equivalent concentrations (f); *p < 0.05 compared to free drugs.

The viability of lung carcinoma cells incubated in the presence of increasing concentrations of the investigated drugs or drug–polymer nanoparticles in the drug–equivalent concentrations was estimated with the 3-(4,5-dimethylthiazol-2-yl)-2,5-diphenyl tetrazolium bromide (MTT) test after 24 and 48 h of treatment (Fig. 3). The viability of cells incubated without any treatment was taken as 100% (control). No effect of D-*g*-PAAan nanoparticles on lung cell viability was detected (data are not shown), while the concentration- and time-dependent toxic effects of the free drugs were observed. All studied lung cell lines exhibited higher sensitivity to Dox as compared to Cis. Under the action of 0.25, 1.0 and 3.7 μM Dox the viability of LLC cells at 24 h was decreased to 87 ± 7, 65 ± 5 and 26 ± 2%, respectively (Fig. 3c), while 5, 20 and 40 μM Cis decreased the LLC cell viability at 24 h to 87 ± 6, 77 ± 5 and 65 ± 5% only, respectively (Fig. 3d). The A549 cells exhibited a viability decrease to 92 ± 6, 89 ± 5 and 81 ± 5% at 24 h after treatment with 1.85, 3.7 and 7.4 μM Dox, correspondingly (Fig. 3e), while 5, 20 and 40 μM Cis had an inhibiting effect towards A549 cell viability that was detected at the level of 83 ± 6%, 78 ± 5% and 75 ± 5% at 24 h, respectively (Fig. 3f).

**Fig. 4:**
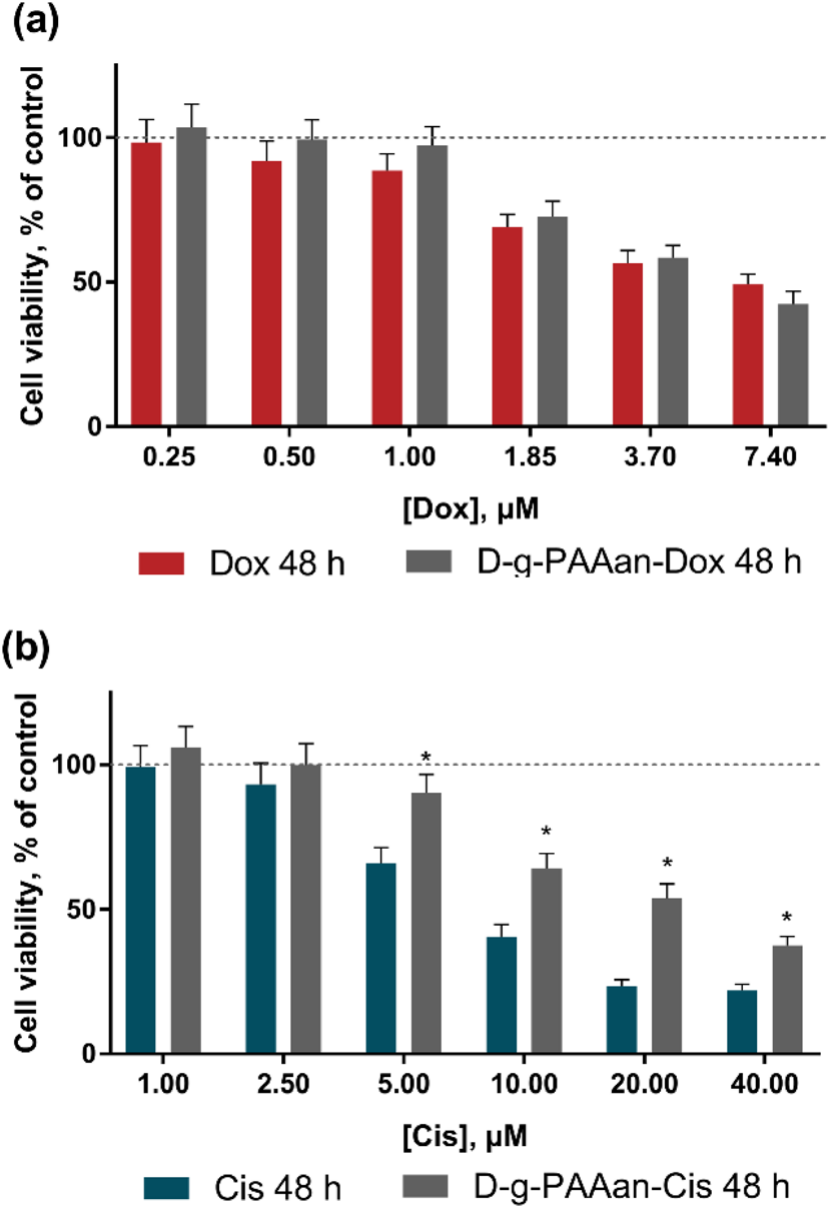
Viability of the normal human lung fibroblast HEL299 cells, treated for 48 h with: (a) – free Dox or D-*g*-PAAan-Dox in Dox-equivalent concentrations, (b) – free Cis or D-*g*-PAAan-Cis in Cis-equivalent concentrations; *p < 0.05 compared to free drugs.

A marked effect of drug encapsulation within D-*g*-PAAan nanoparticles was revealed on LLC and A549 cell viability. LLC cells were found to be more sensitive to D-*g*-PAAan-Dox that at a 1.85 μM Dox concentration the viability decreased to 31% at 24 h as compared with the free drug (Fig. 3c), whereas A549 cell viability was decreased to 34% at 48 h under the action of D-*g*-PAAan-Dox at a 3.70 μM Dox concentration as compared with that of the free drug (Fig. 4e). The D-*g*-PAAan nanoparticles had a prolonged effect on the toxicity of Cis against human and murine LLC (Fig. 3c–f). Thus, D-*g*-PAAan-Cis at 48 h decreased the viability of LLC and A549 cells on 26 and 21% as compared with the free Cis effect correspondingly at a 20 μM equivalent Cis concentration (Fig. 3d and f). Treatment of cells with drug-D-*g*-PAAan nanoparticles was followed by a significant drop in IC_50_ for the D-*g*-PAAan-loaded drugs (Table 2). Visual changes in cell quantity and morphology were also observed with phase-contrast microscopy. As shown in Fig. 4a and b, complexation of Dox and Cis with D-*g*-PAAan nanoparticles caused a decrease in viable cells at 24 h. These data denote D-*g*-PAAan nanoparticles’ ability to potentiate both Dox and Cis toxic effects against cancer cells.

**Table 2:** Half-maximal inhibitory concentration (IC_50_) of free drugs and drug-loaded D-*g*-PAAan nanoparticles on cell viability

	LLC cells		A549 cells	
IC_50_, μM	24 h	48 h	24 h	48 h
Dox	1.8 ± 0.3	0.9 ± 0.2	19.1 ± 3.9	7.4 ± 3.1
D-*g*-PAAan-Dox	0.9 ± 0.2^a^	0.5 ± 0.1^a^	5.6 ± 2.4^a^	2.2 ± 1.2^a^
Cis	68 ± 2	34 ± 2	97 ± 3	23 ± 2
D-*g*-PAAan-Cis	33 ± 2^a^	12 ± 1.2^a^	60 ± 2^a^	8 ± 2.2^a^

a (*p* ≤ 0.05 compared to free drugs).

In contrast, the normal human lung fibroblast HEL299 cells exhibited similar sensitivity to both free and D-*g*-PAAan-loaded Dox (Fig. 4a). HEL299 cell viability was decreased to 58 ± 4 and 57 ± 4% under treatment with 3.7 μM free and D-*g*-PAAan-loaded Dox, respectively. The IC_50_ value of Dox was not significantly changed after its complexation with D-*g*-PAAan nanoparticles (Table 3). For Cis, D-*g*-PAAan nanoparticles were found to even protect HEL299 cells against free Cis cytotoxicity (Fig. 4b). Free and D-*g*-PAAan-loaded Cis at 20 μM decreased the HEL299 cell viability to 23 ± 2 and 54 ± 5%, respectively. Thus, the IC_50_ value for the D-*g*-PAAan-loaded Cis was increased by 2.6 times as compared with that of free Cis (Table 3).

**Table 3:** IC_50_ of free drugs and drug-loaded D-*g*-PAAan nanoparticles on HEL299 cell viability

IC_50_, μM	Dox	D-*g*-PAAan-Dox	Cis	D-*g*-PAAan-Cis
48 h	6 ± 1	5 ± 1	9 ± 2	23 ± 4^a^

a (*p* ≤ 0.05 compared to free drugs).

It was shown that the studied D-*g*-PAAan nanoparticles increased toxic potential of the drugs against human and mouse lung carcinoma cells (Fig. 3c–f), but not against normal human lung cells (Fig. 4). The observed selective biological effects of anticancer drug toxicity upon their encapsulation in the D-*g*-PAAan nanoparticles suggest different modes of interaction between the developed nanoparticles and cancer cells in comparison to normal cells.

### Intracellular ROS generation

**Fig. 5:**
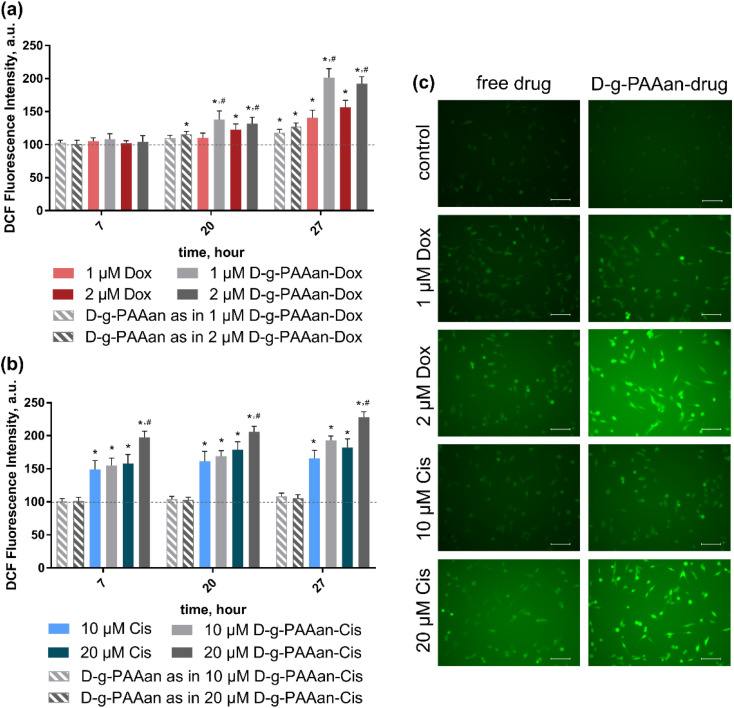
ROS generation in LLC cells, incubated with: (a) either free Dox or free D-*g*-PAAan at concentrations used in D-*g*-PAAan-Dox nanoparticles (1 and 2 μM); (b) either free Cis or free D-*g*-PAAan at concentrations used in D-*g*-PAAan-Cis nanoparticles (10 and 20 μM); *p < 0.05 compared to control; #*p* < 0.05 compared to free drugs; (c) the fluorescence microscopy images of cells after the treatment with the respective drug formulation and further 55 min incubation with DCFH-DA: the scale bar corresponds to 20 μm.

Oxidative stress induction presents a promising anticancer strategy due to the high sensitivity of cancer cells to the ROS level increase^28,29^. Extranuclear effects of both Dox and Cis include ROS production induction^30,31^. The intracellular level of generated ROS in LLC cells after 7, 20 and 27 h of exposure to free and D-*g*-PAAan-loaded Dox and Cis was estimated using an oxidative-sensitive fluorescence dye 2,7-dichlorofluorescin diacetate (DCFH-DA) both quantitively and qualitatively (Fig. 5). The ROS generation of the untreated LLC cells was set as 100%.

Increasing the concentrations of both free drugs administered to the cells provoked a time- and dose-dependent increase in intracellular ROS generation in LLC cells (Fig. 5) that denoted their prooxidant activities^32^. Thus, free 2 μM Dox and 20 μM Cis increased ROS levels in LLC cells to 156 ± 11 and 182 ± 13% at 27 h of incubation, correspondingly (Fig. 5a and b). However, once Dox and Cis were loaded into D-*g*-PAAan particles, a further strong increase in DCF fluorescence intensity revealed the oxidant stress escalation. Thus, 2 μM D-*g*-PAAan-Dox and 20 μM D-*g*-PAAan-Cis increased ROS levels in LLC cells to 192 ± 10 and 228 ± 8% after 27 h of incubation, correspondingly (Fig. 5a and b). In parallel the detected increase in the green fluorescence intensity was observed with fluorescence microscopy (Fig. 5c). Thus, the obtained data suggested that drug-loaded D-*g*-PAAan-nanoparticles promoted oxidative stress in LLC cells by increasing intracellular ROS generation.

### Caspase 3/7 activity

**Fig. 6:**
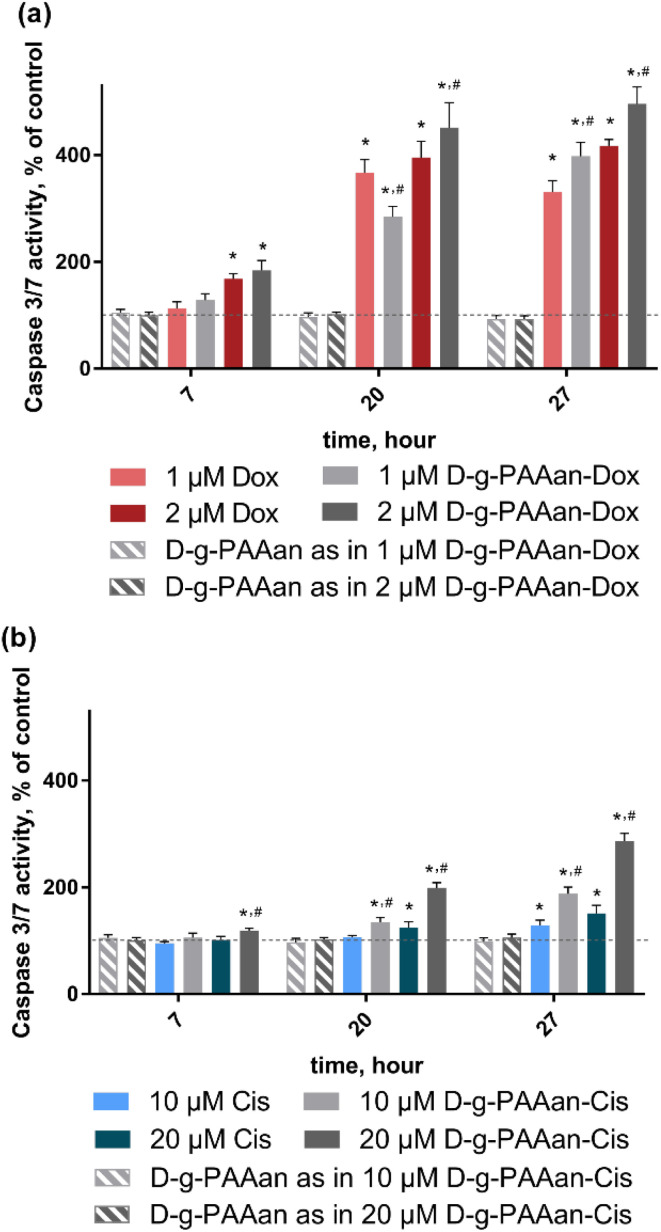
Caspase 3/7 activity in LLC cells, incubated with: (a) either free Dox, free D-*g*-PAAan or D-*g*-PAAan-Dox at concentrations used in D-*g*-PAAan-Dox nanoparticles (1 and 2 μM Dox); (b) either free Cis, free D-*g*-PAAan or D-*g*-PAAan-Cis at concentrations used in D-*g*-PAAan-Cis nanoparticles (10 and 20 μM Cis); *p < 0.05 compared to control; #*p* < 0.05 compared to free drugs.

ROS are increasingly recognized as important initiators and mediators of apoptosis^33^ suggesting that the developed nanoparticles could finally activate the caspase cascade. Therefore, we determined whether the observed cell viability decrease and oxidative stress in LLC cells upon treatment with drug-loaded D-*g*-PAAan nanoparticles were associated with caspase-3/7 activation. Caspase 3/7 activity of untreated LLC cells was set as 100%. The D-*g*-PAAan nanoparticles themselves had no effect on the caspase-3/7 activity, whereas the treatment with both free drugs resulted in a time-dependent increase of the enzyme activity up to 417 ± 12 and 151 ± 15% for 2 μM Dox and 20 μM Cis at 27 h (Fig. 6). Once the drugs were encapsulated in the D-*g*-PAAan nanoparticles, a further increase in the caspase-3/7 activity in LLC cells was found. LLC cells were characterized with caspase-3/7 activity at the levels of 496 ± 32 and 287 ± 14% of the control after 27 h of treatment with D-*g*-PAAan nanoparticles loaded with 2 μM Dox and 20 μM Cis, respectively (Fig. 6). These finding suggested that both Dox- and Cis possessed higher proapoptotic potential when encapsulated in the D-*g*-PAAan nanoparticles.

### Therapeutic effect on a 3D LLC cell spheroid model

A three-dimensional (3D) cell culture is realised with the generation of multi-cellular spheroids. These *in vivo*-like cell aggregates promote direct cell–cell interaction, and thus recapitulate the structures and functions of the native organs and tissues. In cancer research, multi-cellular spheroids simulate intact human tumors featuring similar tissue architectures. Thus, the behavior of 3D-cultured cells is more reflective of *in vivo* cellular responses and can more accurately produce information about pharmacological activity.^34,35^

**Fig. 7:**
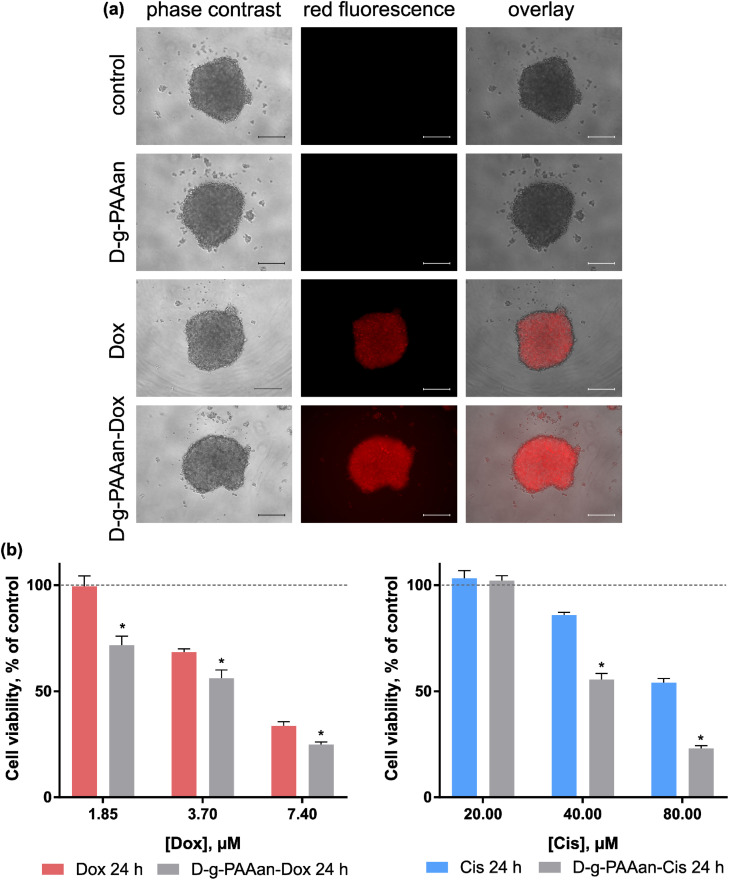
LLC cell 3D spheroids treated with free drugs and drug-nanocomplexes: microscopy of LLC cell 3D spheroids, treated for 24 h with free Dox or D-*g*-PAAan-Dox in 7.4 μM Dox-equivalent concentration (a), scale bar 250 μm; LLC cell 3D spheroid viability after treatment for 24 h with the free drug or D-*g*-PAAan-drug in drug-equivalent concentrations (b), *p < 0.05 compared to the free drug.

To investigate a potential correlation of the enhanced toxic effect of D-*g*-PAAan nanoparticles with a more effective intracellular drug accumulation, the cellular uptake of free Dox and D-*g*-PAAan-Dox was first studied. Since Dox possesses strong absorption and fluorescence in the visible spectral region, tracking of Dox-loaded nanoparticles is possible with non-invasive direct fluorescent-based techniques. 3D LLC cell spheroids were incubated in the presence of 7.4 μM Dox or D-*g*-PAAan-Dox in a drug-equivalent concentration, examined with phase-contrast and fluorescence microscopy. The fluorescence microscopy images illustrate that D-*g*-PAAan-Dox nanoparticles were internalized faster than the free drug as evidenced by brighter intracellular fluorescence (Fig. 7a). The observed improved accumulation of Dox suggested the effective accumulation of the D-*g*-PAAan nanoparticles in LLC spheroids that could be linked to the interplay of the surfaces of the nanoparticles and cells in the 3D environment at a relatively high Dox concentration. The functional groups of the polymer were partially bonded with Dox molecules that could result in charge shielding in the grafted chains and shrinking the D-*g*-PAAan coil at lower pH. The observed accumulation increase of Dox upon D-*g*-PAAan nanoparticle delivery pointed to the perspective application of the developed nanoparticles for targeted drug delivery and will be investigated further in animal models in the near future.

To assess the possible therapeutic effect on the 3D LLC cell spheroid model cell viability was estimated after 24 h of treatment with increasing doses of free and D-*g*-PAAan-Dox. Free D-*g*-PAAan had no effect on the viability of 3D LLC cell spheroids, while Dox treatment of cell spheroids resulted in dose-dependent cell viability decrease (Fig. 7b). Thus, free 1.85, 3.70 and 7.40 μM Dox inhibited the viability of 3D LLC cell spheroids to 99.4 ± 5.0, 68.4 ± 1.6 and 33.6 ± 2.0%, respectively. Once Dox was loaded into D-*g*-PAAan its higher cytotoxicity was detected as compared with the free drug: 1.85, 3.70 and 7.40 μM D-*g*-PAAan-Dox inhibited the viability of 3D LLC cell spheroids to 71.7 ± 4.3, 56.1 ± 4.0 and 24.9 ± 1.2%, correspondingly (Fig. 7a). The encapsulation of Cis into D-*g*-PAAan nanoparticles had a similar effect. The cytotoxicity of Cis towards 3D LLC cell spheroids was increased as compared with that of the free drug at an equivalent concentration (Fig. 7b). The chemotherapeutic drug cytotoxicity modulating effect upon its delivery with D-*g*-PAAan nanoparticles was proved in a 3D multi-cellular spheroid model that suggested its further promising development for cancer treatment.

## Experimental

### Chemicals

Dox and Cis (Sigma-Aldrich, Co, Ltd, USA) were dissolved in distilled water with a maximum concentration of 40 and 500 μg mL^−1^, correspondingly.

Dulbecco’s modified eagle medium (DMEM) liquid medium, phosphate buffered saline (PBS), penicillin/streptomycin, l-glutamine, and trypsin were obtained from PAN-Biotech (Aidenbach, Germany). Eagle’s minimum essential medium (EMEM) was purchased from CLS cell lines service (Eppelheim, Germany). Fetal bovine serum (FBS), 3-(4,5-dimethylthiazol-2-yl)-sodium acetate anhydrous and MTT were obtained from Sigma-Aldrich Co. (St-Louis, USA). Acetic acid, methanol, acetonitrile, formic acid and trypan blue were from Carl Roth GmbH + Co. KG (Karlsruhe, Germany).

### Polymer nanocarrier synthesis

The D-*g*-PAA copolymer was synthesized by the radical graft polymerization method using a Ce(iv)/HNO_3_ redox system. PAA was grafted on certified dextran with a molecular weight of *M*_w_ = 7 × 10^4^ g mol^−1^ (produced by Serva). The synthesis and identification of the sample are described in detail in ref. ^36^.

**Fig. 8:**
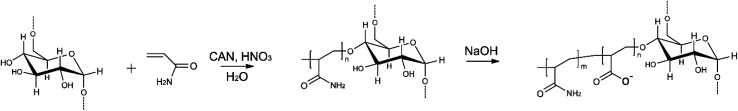
Polymer nanocarrier synthesis: radical graft polymerization method with a Ce(iv)/HNO_3_ redox system and grafting on certified dextran and alkaline hydrolysis.

The branched anionic polyelectrolyte (D-*g*-PAAan) used as a carrier for Dox or Cis molecules was obtained by alkaline hydrolysis of the synthesized copolymer D-*g*-PAA. The hydrolysis of the polymer and the features of the molecular structure of branched polyelectrolytes are described in detail in ref. ^21^. D-*g*-PAAan used in the current work has 32% of hydrolyzed functional groups. The molecular parameters of the copolymer in anionic form were: *M*_w_ = 2.15 × 10^6^ g mol^−1^ and *M*_w_/*M*_n_ = 1.72. The scheme of synthesis of the D-*g*-PAAan copolymer in anionic form is shown in Fig. 8.

Stock solution of the D-*g*-PAAan copolymer (1000 μg mL^−1^) was prepared in distilled water. The D-*g*-PAAan copolymer and Dox or Cis were mixed in a 1 : 1 volume ratio for obtaining water-soluble D-*g*-PAAan + Dox (500 + 40 μg mL^−1^) or D-*g*-PAAan + Cis (500 + 250 μg mL^−1^) nanoparticles, correspondingly. The stock concentrations of Dox and Cis in D-*g*-PAAan-Dox and D-*g*-PAAan-Cis nanoparticles were 74 and 830 μM, respectively.

### Dynamic light scattering

Particle size distribution was evaluated with a Zetasizer Nano S (Malvern Instruments, UK) equipped with a He–Ne laser (633 nm). Data were recorded at room temperature in backscattering modus at a scattering angle of 2*θ* = 173°.

Zeta potential measurements were used for ascertaining the electrokinetic potential of particles in aqueous solution. Measurements were conducted on a Zetasizer Nano-ZS90 (Malvern, Instruments, UK) at room temperature.

### Drug release

D-*g*-PAAan-Dox and D-*g*-PAAan-Cis nanoparticles (1000 μL) were added to a dialysis bag (MWCO 1 kDa, Spectra/Por® Float-A-Lyzer® G2 (Carl Roth GmbH + Co. KG, Karlsruhe, Germany). The dialysis system was suspended in a release volume of 100 mL buffer at 37 °C and rotated at 200 rpm (1 : 100 dilution between donor and acceptor compartments). At scheduled intervals, 500 μL of the release medium was collected for the HPLC-ESI-MS/MS analysis.

The same volume of fresh buffer at the same temperature was added immediately to maintain a constant release volume. Acetate and PBS buffers were used for pH 5.0 and 7.4, respectively. The obtained data were normalized with the buffer control and expressed as a % of the respective control sample, analyzed at 0 h. The Dox concentration was assessed with the HPLC-ESI-MS/MS analysis described before,^37^ whereas Cis quantification required the establishment of a new method, described below.

**Fig. 9:**
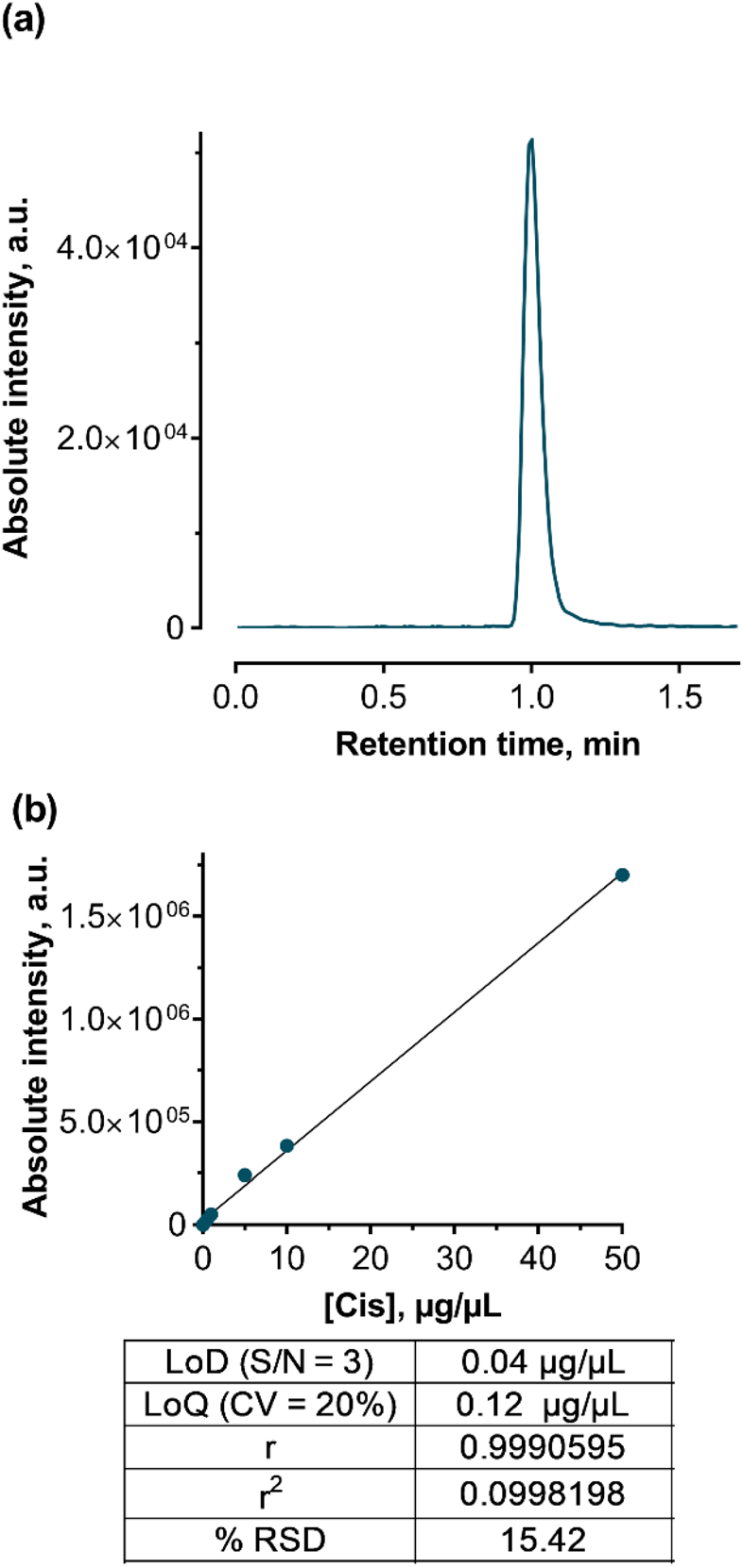
Data of the developed HPLC-ESI-MS/MS method for Cis detection and quantification: a representative MRM-chromatogram of Cis (a) and a calibration curve with the method's performance characteristics used for drug content quantification: LOD – limit of detection, S/N – signal/noise ratio, LOQ – limit of quantitation, and RSD – relative standard deviation (b).

Elution and separation of Cis were performed using an Eclipse XDB-C18 column (4,6 × 100 mm) with a 3 μm particle size under isocratic conditions with a mobile phase of methanol and a 0.1% formic acid water solution. The flow rate was set at 0.5 mL min^−1^. The chromatographic reverse phase conditions and optimized MS/MS parameters are presented in Table 4. For identification and quantification, the Cis adduct ion with Na [M^+^Na]^+^ was chosen (Fig. 9a).

**Table 4:** HPLC-ESI-MS/MS conditions for analysis of Cis

Chromatographic conditions	
Column and its temperature	MN nucleodor EC100/3C18, 40 °C
Mobile phase	Methanol: 0.1% formic acid H_2_O
Flow rate	0.5 mL min^−1^
Run time	1.7 min
Injection volume	3 μL

MS/MS conditions	
Desolvation line temperature	250 °C
Heat block temperature	400 °C
Target molecular ion	332.9 [M^+^Na]^+^*m*/*z*
Product ions	305.9, 23.05 *m*/*z*
Time window	0–1.7 min
Dwell time	0.2 s
Interface voltage	4.5 kV
Nebulizing gas flow	3 L min^−1^
Drying gas flow	15 L min^−1^

HPLC-ESI-MS/MS analysis was performed in positive mode with the usage of multiple reactions monitoring (MRM) mode, which provides the best sensitivity and accuracy of measurements. After MS/MS optimization, a unique MRM-transition that includes a precursor and two characteristic product ions was acquired and used for further identification and quantification. The ionized Cis adduct with Na ([M + Na]^+^, 332.9 *m*/*z*) was used as a precursor ion with the most abundant fragment ions of 305.9 and 23.05 *m*/*z*.

Cis calibration standards from 0.1 to 50 μg mL^−1^ were prepared from a 250 μg mL^−1^ water stock solution. These standards were stored in the dark at 4 °C. Quantification was achieved using the regression curve (Fig. 9b) according to the linear regression eqn (1):

1 *y* = 33616.0*x* + 2886.2

The obtained data were normalized with the buffer controls and expressed as a percentage of the respective control sample, analyzed at 0 h.

### 2D cell culture

LLC cells were kindly supplied by the bank of cell cultures and transplantable experimental tumors of the R.E. Kavetsky Institute of Experimental Pathology, Oncology and Radiobiology, NAS of Ukraine (Kyiv, Ukraine). Lung carcinoma human A549 and normal human lung fibroblast HEL299 cell lines were purchased from AddexBio Technologies (San Diego, CA, USA). Cells were maintained in a 5 mL DMEM (LLC and A549) or EMEM (HEL 299) medium supplemented with 10% FBS, 1% penicillin/streptomycin and 2 mM glutamine, using 25 cm^2^ flasks from Sarstedt (Nümbrecht, Germany) at 37 °C with 5% CO_2_ in a humidified incubator binder (Tuttlingen, Germany). Passaging was performed once cells reached ≈80%. Treatment with trypsin (1 : 10 in PBS) was used to detach adherent cells. The number of the viable cells was counted with the use of a Roche Cedex XS Analyzer (Basel, Switzerland) after staining with 0.1% trypan blue.

### 2D cell viability

Cells (10^4^ cells per well), cultured in 96-well cell culture plates by Sarstedt (Nümbrecht, Germany) for 24 h, were treated with a 1% FBS DMEM medium containing 0–7.4 μM Dox and D-*g*-PAAan-Dox, 0–40 μM Cis and D-*g*-PAAan-Cis in a drug equivalent concentration. Cells were observed with a Keyence BZ-9000 BIOREVO microscope (Osaka, Japan) in combination with the Keyence BZ-II viewer acquisition software (Osaka, Japan). Cell viability was determined with an MTT reduction assay^38^ at 24 and 48 h. Briefly, cells were incubated for 2 h at 37 °C in the presence of 0.5 mg mL^−1^ MTT. Diformazan crystals were dissolved in DMSO and determined at 570 nm with a microplate reader Tecan Infinite M200 Pro (Männedorf, Switzerland).

### Intracellular ROS generation

To determine ROS production DCFH-DA (Sigma-Aldrich Co., St-Louis, USA) was applied. A 5 mM stock solution of DCFH-DA was prepared in DMSO, stored at −20 °C and diluted with PBS immediately before use. LLC cells were seeded into 96-well plates (10^4^ cells per well) and incubated for 24 h. Then the medium was exchanged with the one containing free Dox or D-*g*-PAAan-Dox in 1 and 2 μM Dox-equivalent concentrations and cells were incubated under normal cell culture conditions for 7, 20 and 27 h. At given time points cells were washed once with PBS. DCFH-DA (5 μM) was added and the fluorescence intensity (*λ*_ex_ = 488 nm, *λ*_em_ = 520 nm) was recorded at 45 min with the microplate reader Tecan Infinite M200 Pro (Männedorf, Switzerland). At 55 min of incubation fluorescence images of cells were obtained with the fluorescence microscope Keyence BZ-9000 BIOREVO (Osaka, Japan), equipped with a green filter (*λ*_ex_ = 472 nm, *λ*_em_ = 520 nm).

### Caspase 3/7 activity

LLC cells were seeded into 96-well plates (10^4^ cells per well) and incubated for 24 h. The cells were treated with free Dox or D-*g*-PAAan-Dox in 1 and 2 μM Dox-equivalent concentrations for 7, 20 and 27 h. The activity of caspase 3/7 was determined using the Promega Caspase-Glo® 3/7 activity assay kit (Madison, USA) according to the manufacturer’s instructions. Briefly, plates were removed from the incubator and allowed to equilibrate to room temperature for 30 min. After treatment, an equal volume of the Caspase-Glo 3/7 reagent containing a luminogenic peptide substrate was added, followed by gentle mixing with a plate shaker at 300 rpm for 1 min. The plate was then incubated at room temperature for 2 h. The luminescence intensity of the products of the caspase 3/7 reaction was measured with the microplate reader Tecan Infinite M200 Pro (Männedorf, Switzerland).

### 3D cell spheroid generation

The LLC cell spheroids were generated in Corning® ultra-low attachment U-bottomed (round) 96-well plates.

Briefly, LLC cells were seeded in a serum-free culture DMEM medium (10^4^ cells per well), shortly centrifuged and then placed in the incubator Binder (Tuttlingen, Germany) at 37 °C, 5% CO_2_, and 95% humidity.

### 3D cell spheroid viability

After spheroid formation (at 24 h after seeding), the agents under the study were added: D-*g*-PAAan, 0–7.4 μM Dox and D-*g*-PAAan-Dox, 0–80 μM Cis and D-*g*-PAAan-Cis in a drug equivalent concentration. At 24 h the 3D cell viability was estimated with Promega CellTiter-Glo® 3D Cell Viability Assay (Promega, Madison, USA). Briefly, the plate was removed from the incubator and allowed to equilibrate to room temperature for 30 min. An equal volume of the CellTiter-Glo® 3D reagent containing a luminogenic peptide substrate was added, followed by gentle mixing with a plate shaker at 300 rpm for 5 min. The plate was then incubated at room temperature for 25 minutes to stabilize the luminescent signal. The luminescence intensity of the products was measured with the microplate reader Tecan Infinite M200 Pro (Männedorf, Switzerland).

### Fluorescence microscopy

After spheroid formation (at 24 h after seeding), the agents under the study were added: D-*g*-PAAan, Dox and D-*g*-PAAan-Dox in a 7.4 μM Dox equivalent concentration. Then, 3D LLC cell spheroid were treated for 7 h and visualized with a fluorescence microscope Keyence BZ-9000 BIOREVO (Osaka, Japan) equipped with a red (*λ*_ex_ = 480 nm, *λ*_em_ = 600 nm) filter and the respective acquisition software Keyence BZ-II Viewer (Osaka, Japan). The overlayed images were processed with the Keyence BZ-II Analyzer Software (Osaka, Japan).

### Statistics

All experiments were carried out with a minimum of four replicates. Data analysis was performed using the GraphPad Prism 7 Software (GraphPad Software Inc., USA). Paired Student’s *t*-test was performed. Differences with *p*-values <0.05 were considered to be significant.

The half-maximal inhibitory concentration (IC_50_) value was calculated with specialized software GraphPad Prism 7 (GraphPad Software Inc.). Individual concentration–effect curves were generated by fitting the logarithm of the compound concentration *versus* the corresponding normalized cell viability using nonlinear regression.

## Conclusions

The polyacrylamide polymer was synthesized by the radical graft polymerization method, grafted on a certified dextran polymer and alkaline-hydrolyzed to get a star-like branched anionic dextran-*graft*-polyacrylamide (D-*g*-PAAan) copolymer nanoparticle. For this, the commonly used chemotherapeutic drugs Dox and Cis were encapsulated within D-*g*-PAAan nanoparticles, whose size was estimated to be 80 nm. The obtained Dox- or Cis-containing D-*g*-PAAan nanoparticles released drugs in response to a pH decrease to 5.0 that pointed towards the opportunity for pH-triggered drug delivery. D-*g*-PAAan nanoparticles increased the toxic potential of the drugs against human and mouse lung carcinoma cells (A549 and LLC), but not against normal human lung cells (HEL299). The drug-loaded D-*g*-PAAan-nanoparticles promoted further oxidative stress and apoptosis induction in LLC cells by increasing intracellular ROS generation and activation of caspase 3/7. The observed selective cytotoxic effects of anticancer drug toxicity upon their encapsulation in the D-*g*-PAAan nanoparticles suggested differential interactions between the developed nanoparticles and the cancer or normal cells, respectively, that pointed to a promising approach for targeted cancer treatment.

The data obtained in the study indicate that the strategy of chemotherapeutic drug encapsulation within the D-*g*-PAAan nanoparticle allows not only to realize pH-triggered drug release but also to potentiate its cytotoxic, prooxidant and proapoptotic effects against lung carcinoma cells *in vitro*. As a pH decrease is observed in most solid tumors, the proposed drug-delivery polymer nanoparticle responsive to the slightly acidic extracellular pH environment of solid tumors provides a promising approach for cancer treatment.

## Author contributions

Conceptualization: MF and YP, data curation: AG, formal analysis: AG, funding acquisition: AG and MF, investigation: AG, SvP, StP, PV, VC, and UR, methodology: AG and SG, project administration: AG, resources: YP and MF, software: AG, supervision: MF, NK, and YP, validation: AG and SG, visualization: AG and SG, writing – original draft: AG, NK, and YP, writing – review & editing: AG, YP, and MF.

## Conflicts of interest

There are no conflicts to declare.

## Supplementary Material
